# Blocking VCAM-1 Prevents Angiotensin II-Induced Hypertension and Vascular Remodeling in Mice

**DOI:** 10.3389/fphar.2022.825459

**Published:** 2022-02-10

**Authors:** Liangqingqing Yin, Jie Bai, Wei-Jia Yu, Ying Liu, Hui-Hua Li, Qiu-Yue Lin

**Affiliations:** ^1^ Institute of Cardiovascular Diseases, First Affiliated Hospital of Dalian Medical University, Dalian, China; ^2^ Department of Emergency Medicine, Beijing Key Laboratory of Cardiopulmonary Cerebral Resuscitation, Beijing Chaoyang Hospital, Capital Medical University, Beijing, China

**Keywords:** VCAM-1, hypertension, macrophage adhesion, inflammation, oxidative stress, vascular dysfunction

## Abstract

Adhesion of monocytes to the vascular endothelium frequently leads to an inflammatory response, which contributes to hypertension and vascular remodeling. Vascular cellular adhesion molecule-1 (VCAM-1) plays an important role in leukocyte adhesion and migration during inflammatory diseases. However, its role in angiotensin (Ang) II -induced hypertension and vascular dysfunction remains largely unknown. Wild-type (WT) mice were administered a VCAM-1 neutralizing antibody (0.1 or 0.2 mg/mouse/day) or IgG control and then infused with Ang II (490 ng kg^−1^ min^−1^) or saline continuously for 14 days. Systolic blood pressure (SBP) was measured with a tail-cuff system, pathological changes in the aorta were assessed by histological staining, and vascular relaxation was analyzed an aortic ring assay. Our results indicated that compared with saline infusion, Ang II infusion significantly upregulated VCAM-1 expression in the mouse aorta and serum. Moreover, Ang II infusion markedly increased arterial hypertension, wall thickness, fibrosis, infiltration of Mac-2^+^ macrophages, reactive oxygen species (ROS) production and vascular relaxation dysfunction. Conversely, blockade of VCAM-1 with a neutralizing antibody substantially alleviated these effects. *In vitro* experiments further confirmed that the VCAM-1 neutralizing antibody inhibited Ang II-induced macrophage adhesion and migration and DNA damage and oxidative stress in endothelial cells (ECs). In conclusion, these results indicate that blockade of VCAM-1 exerts a protective effect against Ang II-induced arterial hypertension and dysfunction by regulating monocytes adhesion and infiltration into the endothelium and represents a novel therapeutic approach for hypertension.

## Introduction

Hypertension is the primary risk factor for various cardiovascular diseases (CVDs), which are associated with high morbidity and mortality worldwide (Dorans et al., 2018). Blood pressure (BP) can be modulated by multiple physiological systems, including the nervous system, the renin-angiotensin-aldosterone system (RAS) and the endothelin system, and aging and environmental factors, such as high salt intake, which independently or jointly contribute to endothelial dysfunction and the development of various CVDs (Caillon et al., 2019). The RAS has emerged as one of the most relevant factors in the occurrence and development of CVDs. Angiotensin (Ang) II, as a main peptide of the RAS, can stimulate vascular endothelial cells (ECs) to express cytokines, chemokines, growth factors and adhesion molecules, which are vital for regulating vascular pathophysiological processes, such as alterations in arterial tone, inflammatory responses, fibrosis, and extracellular matrix remodeling, through angiotensin type 1 receptor (AT1R) (Ruiz-Ortega et al., 2001). Experimental findings indicate that Ang II can promote monocyte but not neutrophil infiltration and maturation into proinflammatory macrophages and induce vascular dysfunction and arterial hypertension (Wenzel et al., 2011). Our previous data suggest that Ang II upregulates the expression of the chemokine CXCL1, which promotes the migration of CXCR2^+^ monocytes/macrophages to vessels, resulting in the initiation and progression of hypertension and vascular remodeling (Wang et al., 2016). Our recent study demonstrated that the adhesion molecule intercellular adhesion molecule-1 (ICAM-1) plays an important role in Ang II-induced hypertension and vascular dysfunction by promoting LFA-1^+^ monocyte adhesion to the endothelium (Lang et al., 2020), indicating that adhesion molecules are involved in hypertension and vascular dysfunction.

Vascular cellular adhesion molecule-1 (VCAM-1; also known as CD106), a member of the immunoglobulin (Ig) superfamily, is a transmembrane type I protein that is expressed on the cell membrane (Cook-Mills et al., 2011). VCAM-1 is susceptible to proteolytic cleavage near the transmembrane site by tumor necrosis factor-ɑ (TNF-α) converting enzyme, which generates soluble VCAM-1 (sVCAM-1) (Garton et al., 2003). sVCAM-1 is canonically induced in the vascular endothelium by stimulation of proinflammatory cytokines such as TNF-α and interleukin (IL)-1β (Troncoso et al., 2021). Studies have reported that VCAM-1 can interact with α4β1 integrin (VLA-4), which is expressed on the surface of leukocytes and is important for regulating their recruitment, rolling, adhesion, and migration, and the activation of intracellular signaling, which promotes promoting the transmigration of proinflammatory cells from the circulation to the site of tissue damage (Ocaranza et al., 2018; Zhong et al., 2018; Troncoso et al., 2021). Basic experimental and clinical data have indicated that higher levels of VCAM-1 in serum or tissue are associated with coronary artery disease, ischemic cardiomyopathy, acute myocarditis, and atrial fibrillation (AF); thus, VCAM-1 represents a potential biomarker for CVDs (Troncoso et al., 2021). However, the role of VCAM-1 in hypertension and vascular dysfunction remains largely unknown.

In the current study, we investigated whether blockade of VCAM-1 with a VCAM-1 neutralizing antibody prevents Ang II-induced adhesion and migration of macrophages and arterial hypertension. Our results revealed that blocking VCAM-1 with a neutralizing antibody substantially alleviated the Ang II-induced elevation of arterial blood pressure and vascular dysfunction and reduced the adhesion and infiltration of monocytes/macrophages and reactive oxygen species (ROS) production in the aorta. These results indicate that selective blockade of VCAM-1 may represent a novel therapeutic option for hypertension.

## Materials and Methods

### Animals and Treatment

Wild-type (WT) C57BL/6J mice (male, 8–10 weeks) were acquired from The Jackson Laboratory. A hypertensive model was constructed by infusion of Ang II at a dosage of 490 ng kg^−1^·min^−1^ for 14 days as previously described (Wang et al., 2016; Ji et al., 2017; An et al., 2018). WT mice were intraperitoneally injected with 0.1 or 0.2 mg of anti-VCAM-1 neutralizing antibody (BE0027, Bioxcell) or IgG control every 2 days after Ang II infusion as previously reported (Thomas et al., 2011; Chow et al., 2013). The animals were housed under specific pathogen-free (SPF) conditions and fed a regular diet. The experimental processes were authorized by the Institutional Animal Care and Use Committee (IACUC) at the First Affiliated Hospital of Dalian Medical University (AEE20027).

### Systolic Blood Pressure Measurement

Systolic blood pressure (SBP) was measured and recorded with a tail-cuff instrument (BP-98A, Softron, Japan) as previously reported (Wang et al., 2018). Briefly, the mice were placed on a heating pad for at least 30 min. Conscious and calm mice were placed on a fixator, and the tail was fully exposed. Then, the tail-cuff instrument was placed at the base of the tail. SBP and heart rate data were obtained when the waveform was steady. The data were recorded at least 5 times.

### Histopathological Analyses

Mice were anesthetized with 5% isoflurane and euthanized by cervical dislocation following protocols approved by the Temple University IACUC. Aortic tissues were isolated, fixed with 4% paraformaldehyde (Life-iLab, AC28L112, CN) overnight, and embedded in paraffin or OCT after dehydration as previously described (Lang et al., 2020). Paraffin sections (4 μm) were subjected to hematoxylin and eosin (H&E) and Masson’s trichrome staining as previously described (Lang et al., 2020). Frozen sections (8 μm) were subjected to dihydroethidium (DHE) staining as previously reported (Lang et al., 2020). The excitation wavelength of the microscope was set to 535 nm, and the emission wavelength was set to 610 nm. The aortic tissues were subjected to immunohistochemical staining with primary antibodies against VCAM-1 (66294-1-Ig, Proteintech) and mac-2 (ARG66239, Arigo) as previously described (Wang et al., 2018; Lang et al., 2020). Digital pictures were taken at 200× magnification and analyzed with ImageJ software.

### Enzyme-Linked Immunosorbent Assay

The VCAM-1 concentration in mouse serum was evaluated with an enzyme-linked immunosorbent assay (ELISA) kit (Elabscience, E-EL-M1233c) according to the manufacturer’s instructions. The serum Ang II concentration of the mice was analyzed with an ELISA kit (Elabscience, E-EL-H0326c) with a detection limit of 31.25–2,000 pg/ml and a sensitivity of 18.75 pg/ml. Briefly, mice were anesthetized with 5% isoflurane and the carotid artery was fully exposed by the scissors, whole blood was collected of each mouse and allowed to clot at room temperature (RT) for 30 min, and then centrifuged at 3,000 rpm for 10 min at 4°C, the supernatant was collected by a pipettor and added to an enzyme-labeled plate. The absorbance was measured at 450 nm with a full-wavelength enzyme-labeled instrument.

### Analysis of Malondialdehyde and Glutathione Levels

The levels of malondialdehyde (MDA) and glutathione (GSH) in mouse serum were evaluated by an MDA kit (KGT003, Keygenbio) and GSH kit (KGT006, Keygenbio), respectively, according to the manufacturer’s instructions.

### Immunoblot Analysis

Total protein was extracted from fresh aortas with a protein extraction kit (Keygenbio, KGP250) and protease and phosphatase inhibitors. The proteins (25–30 μg) were subjected to SDS–PAGE, transferred onto polyvinylidene fluoride membranes, which were incubated with primary antibodies against VCAM-1 (66294-1-Ig, Proteintech) and GAPDH (WL03413, Wanleibio). The membranes were incubated with a horseradish peroxidase-conjugated secondary antibody (Sino Biological Inc.), and band detection was performed by chemiluminescence. GAPDH was used as an internal control.

### Real-Time PCR Analysis

Total RNA was extracted from aortas or cultured cells using TRIzol (Sangon Biotech, B511311). cDNA was obtained by using the PrimeScript RT Reagent Kit (Yeasen, 11141ES60). The mRNA levels of VCAM-1, collagen I/III, α-smooth muscle actin (α-SMA), IL-1β, IL-6, TNF-α, monocyte chemoattractant protein-1 (MCP-1), NADPH oxidase (NOX)1, NOX2 and NOX4 were measured using SYBR mix (Accurate Biotechnology Co., Ltd., Hunan, AG11701) and a 7500-fast instrument (ABI, United States) as previously described (Wang et al., 2018). The results were normalized to the level of GAPDH. The primer sequences used are listed in Table 1.

**TABLE 1:** Primers used for quantitative real-time PCR analysis.

Gene	Ref Seq	Forward primer (5′-3′)	Reverse primer (5′-3′)
VCAM-1	NM_011693.3	TTG​GGA​GCC​TCA​ACG​GTA​CT	GCA​ATC​GTT​TTG​TAT​TCA​GGG​GA
Collagen I	NM_007742.4	GAG​TAC​TGG​ATC​GAC​CCT​AAC​CA	GAC​GGC​TGA​GTA​GGG​AAC​ACA
Collagen III	NM_009930.2	TCC​CCT​GGA​ATC​TGT​GAA​TC	TGA​GTC​GAA​TTG​GGG​AGA​AT
α-SMA	NM_007392.3	AGC​CAT​CTT​TCA​TTG​GGA​TGG	CCC​CTG​ACA​GGA​CGT​TGT​TA
IL-1β	NM_008361.4	TGC​CAC​CTT​TTG​ACA​GTG​ATG	TGA​TGT​GCT​GCT​GCG​AGA​TT
TNF-α	NM_013693.3	CAG​GCG​GTG​CCT​ATG​TCT​C	CGA​TCA​CCC​CGA​AGT​TCA​GTA​G
MCP-1	NM_011333.3	TAA​AAA​CCT​GGA​TCG​GAA​CCA​AA	GCA​TTA​GCT​TCA​GAT​TTA​CGG​GT
NOX1	NM_172203.2	CCT​GAT​TCC​TGT​GTG​TCG​AAA	TTG​GCT​TCT​TCT​GTA​GCG​TTC
NOX2	NM_007807.5	TTG​TTT​GGT​TAG​GGC​TGA​ATG​T	GCC​AAT​GTT​GAC​CCA​AGG​ATT​TT
NOX4	NM_015760.5	CAG​ATG​TTG​GGG​CTA​GGA​TTG	GAG​TGT​TCG​GCA​CAT​GGG​TA
GAPDH	NM_008084.3	GGT​TGT​CTC​CTG​CGA​CTT​CA	GGT​GGT​CCA​GGG​TTT​CTT​ACT​C

VCAM-1, vascular cell adhesion molecule 1; α-SMA, α-smooth muscle actin; IL-1β, interleukin 1 beta; TNF-α, tumor necrosis factor alpha; MCP-1, monocyte chemoattractant protein-1; NOX1, NADPH, oxidase 1; NOX2, NADPH, oxidase 2; NOX4, NADPH, oxidase 4; GAPDH, glyceraldehyde 3-phosphate dehydrogenase.

### Vascular Relaxation Analysis

The thoracic aortas of mice from each group were isolated, sliced into 4 mm segments and then carefully placed on force transducers (Danish MyoTechnology, DK) in organ chambers. The aortas were activated initially with norepinephrine (NE), followed by graded concentrations of acetylcholine (ACh) or sodium nitrate (SNP) as previously described (Lang et al., 2020).

### Cell Adhesion Assay

Macrophages were obtained from the tibias and femurs of WT mice and cultured in 1,640 medium (Meilunbio, MA0215). Human umbilical vein endothelial cells (HUVECs) were cultured in ECM. The adhesion of macrophages to HUVECs was assessed as previously described (Lin et al., 2019). Briefly, HUVECs were stimulated with Ang II (100 nM) or saline for 24 h and then blocked with anti-VCAM-1 (5 μg/ml) or IgG control for 4 h. Macrophages were tagged with the fluorescent dye PKH-26 and added to the HUVECs at a macrophage to HUVEC ratio of 10:1. After incubation for 1 h, the nonadherent cells were washed away with PBS, and the adherent cells were photographed by microscopy (Olympus, IX73) at 100x magnification.

### Cell Migration Assay

Macrophage migration was evaluated as described in our previous study (Lin et al., 2019). Briefly, HUVECs were cultured and treated as described for the adhesion assay. Macrophages (5 × 10^4^) were added to the upper chamber of a 24-well Transwell plate (8 μm pore, Corning), and HUVEC-conditioned medium was added to the lower chamber of the plate (Guangzhou Jet Bio-Filtration Co., Ltd.). After incubation for 24 h, the migrated cells were fixed and stained with DAPI. Six random areas were selected and photographed with a microscope (Olympus, IX73) at 100x magnification.

### Cell Immunofluorescence Staining

Macrophages were stimulated with Ang II (100 nM) or saline for 24 h and then blocked with anti-VCAM-1 (5 μg/ml) or IgG control for 4 h. Then, conditioned medium was added to monolayer HUVECs, and the cells were cultured for 24 h. Primary antibodies against γ-H2AX (Abcam, 1:100) and p-ataxia telangiectasia mutated (ATM; Santa Cruz, 1:100) were added to the HUVECs and detected with fluorescence-conjugated secondary antibodies (Boster, BA1135). The cell nuclei were stained with DAPI.

### Statistics

All data are expressed as the mean ± standard deviation (M ± SD). Statistical analyses were performed with GraphPad Prism 8.0 software. Independent *t* test was used to analyze the difference between two groups when the data were normally distributed, and the Wilcoxon rank sum test was used when the data were abnormally distributed. One-way ANOVA followed by post hoc Tukey’s multiple comparisons test was used to analyze the differences among multiple groups when the data were normally distributed, and the Kruskal–Wallis test was used when the data were abnormally distributed. *p* < 0.05 was considered statistically significant.

## Results

### Ang II Promotes VCAM-1 Expression in the Aorta and Serum

**FIGURE 1:**
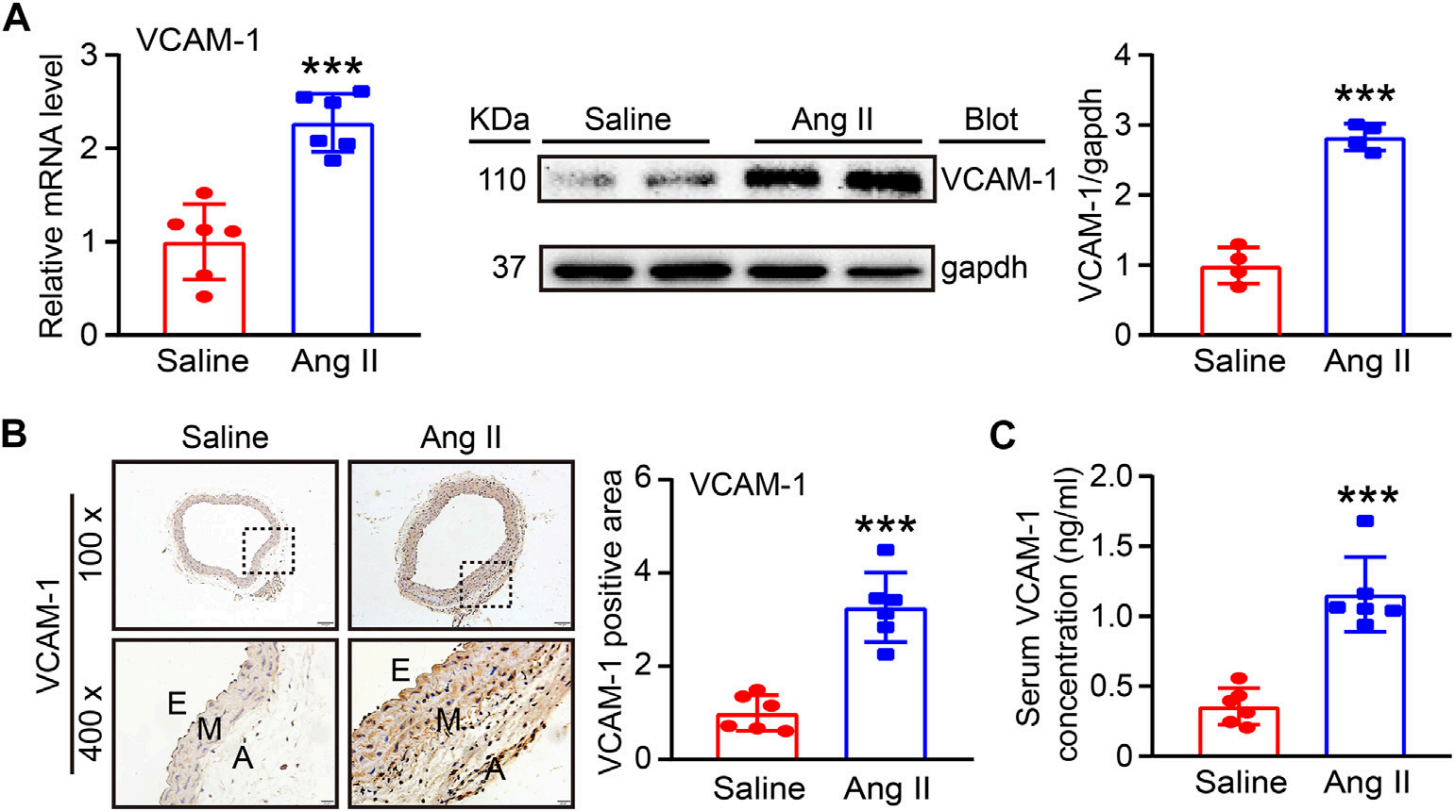
Ang II infusion elevates VCAM-1 levels in the aorta. **(A)** WT mice were infused with Ang II (490 ng kg^−1^ min^−1^) or saline for 14 days. qPCR analysis of the VCAM-1 mRNA level in the aorta (*n* = 6), immunoblot analysis of VCAM-1 expression in the aorta (left) and quantification of the levels of this protein (right, *n* = 4). **(B)** Immunohistochemical staining of arterial tissue with a VCAM-1 antibody at 100x (upper, scale bar = 100 μm) and 400x (lower, scale bar = 20 μm) and quantification of the VCAM-1-positive area (right, *n* = 6). **(C)** Analysis of the VCAM-1 concentration in mouse serum (*n* = 6). The data are expressed as the mean ± SD, and n represents the number of samples. ***p < 0.001 versus saline.

To determine the involvement of VCAM-1 in Ang II-induced hypertension and vascular dysfunction, we first evaluated VCAM-1 levels in the aorta after Ang II infusion for 14 days qPCR and immunoblot analysis revealed that VCAM-1 mRNA and protein levels were significantly higher in Ang II-infused aortas than in saline control-treated aortas (Figure 1A). Immunohistochemical staining further confirmed that VCAM-1 protein expression was upregulated in Ang II-infused aortas (Figure 1B). In addition, ELISA indicated that the serum VCAM-1 level was higher in Ang II-infused mice than in saline-infused controls (Figure 1C). Thus, an increase in VCAM-1 expression may play an important role in the development of Ang II-induced hypertension.

### Blockade of VCAM-1 Alleviates Ang II-Induced Arterial Hypertension, Hypertrophy, and Fibrosis

**FIGURE 2:**
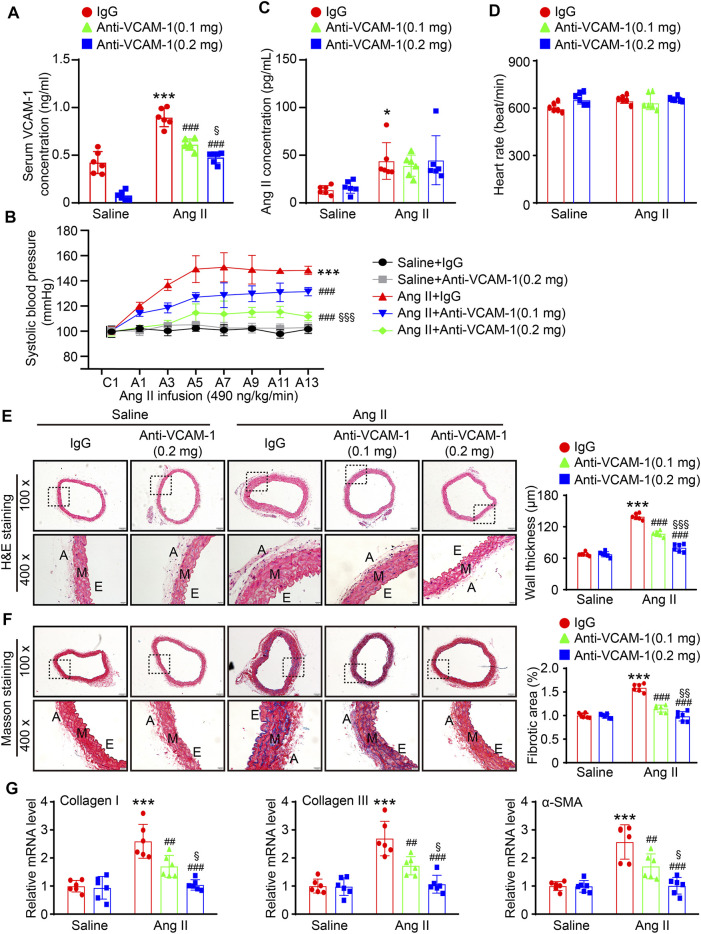
Inhibition of VCAM-1 prevents Ang II-induced hypertension, hypertrophy, and fibrosis in the aorta. **(A)** WT mice were treated with a VCAM-1 neutralizing antibody (0.1 or 0.2 mg/mouse) or IgG control and infused with Ang II (490 ng kg^−1^ min^−1^) or saline for 14 days. Analysis of the serum VCAM-1 concentration in each group (*n* = 6). **(B)** Analysis of aortic SBP during Ang II infusion (*n* = 6). **(C)** Analysis of the serum Ang II concentration in each group (*n* = 6). **(D)** Measurement of the heart rate in each group (*n* = 6). **(E)** H&E staining of the aorta at 100x (upper, scale bar = 100 μm) and 400x (lower, scale bar = 20 μm) and quantification of wall thickness (right, *n* = 6). **(F)** Analysis of collagen deposition in the aorta by Masson staining at 100x (upper, scale bar = 100 μm) and 400x (lower, scale bar = 20 μm) and quantification of the fibrotic area (right, *n* = 6). **(G)** qPCR analysis of collagen I, collagen III and α-SMA mRNA levels (*n* = 6). The data are expressed as the mean ± SD, and *n* represents the number of samples. *p < 0.05 and ***p < 0.001 versus saline; ^##^ *p* < 0.01 and ^###^ *p* < 0.001 versus Ang II + IgG; ^§^ *p* < 0.05, ^§§^ *p* < 0.01 and ^§§§^ *p* < 0.001 versus Ang II + anti-VCAM-1 (0.1 mg).

To further explore the effect of VCAM-1 on hypertension and vascular remodeling, we administered a VCAM-1 neutralizing antibody (0.1 and 0.2 mg/mouse/day) or IgG control to WT mice for 1 day and then administered Ang II (490 ng kg^−1^ min^−1^) or saline via continuous infusion for up to 14 days. Our data showed that the Ang II-induced increases in serum VCAM-1 levels and SBP were dose-dependently reduced in mice administered the VCAM-1 neutralizing antibody (Figures 2A,B), indicating that the VCAM-1 neutralizing antibody effectively blocked VCAM-1 and inhibited hypertension in mice. However, serum Ang II levels and heart rate were similar between the IgG- and anti-VCAM-1-treated mice after saline or Ang II infusion (Figures 2C,D). Moreover, H&E and Masson staining indicated that compared with IgG treatment, administration of the VCAM-1 neutralizing antibody to mice markedly decreased arterial wall thickness and collagen deposition in a dose-dependent manner (Figures 2E,F). Accordingly, compared with IgG, the VCAM-1 neutralizing antibody markedly reduced the Ang II-induced upregulation of the mRNA expression of fibrotic markers, including collagen I, collagen III, and α-SMA, in the aorta (Figure 2G). Interestingly, high dose of anti-VCAM-1 antibody (0.2 mg) had higher attenuation of Ang II-induced increase of SBP and arterial remodeling than low dose of anti-VCAM-1 (0.1 mg) (Figures 2B,E–G).

### Neutralization of VCAM-1 Attenuates Ang II-Induced Macrophage Infiltration and Oxidative Stress

**FIGURE 3:**
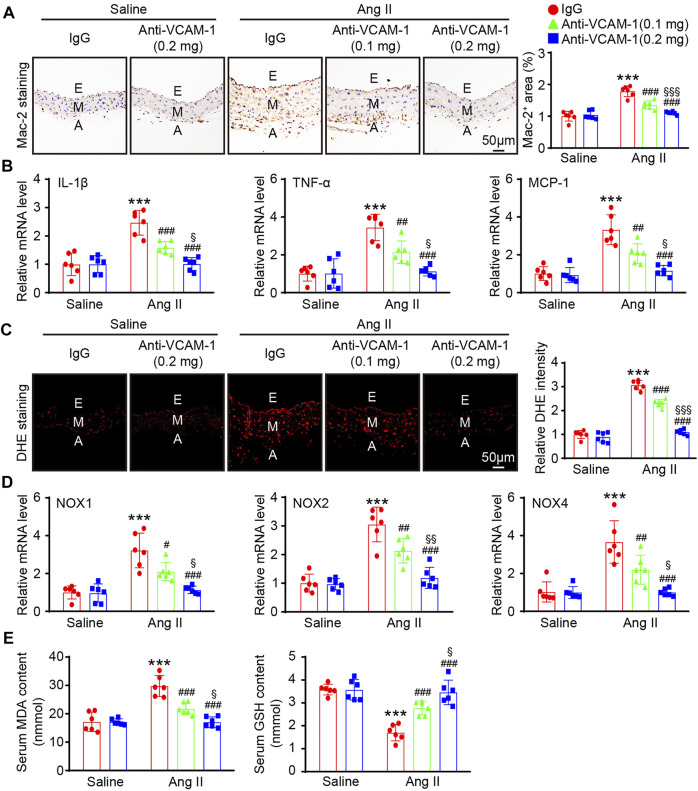
Blockade of VCAM-1 inhibits Ang II-induced macrophage infiltration, inflammation, and oxidative stress. **(A)** WT mice were treated with a VCAM-1 neutralizing antibody (0.1 or 0.2 mg/mouse) or IgG control and infused with Ang II (490 ng kg^−1^ min^−1^) or saline for 14 days. Immunohistochemical staining of the aorta with a Mac-2 antibody (left, scale bar = 50 μm) and analysis of the Mac-2-positive area (right, *n* = 6). **(B)** qPCR analysis of IL-1β, TNF-α and MCP-1 mRNA levels in the aorta (*n* = 6). **(C)** DHE staining of the aorta (left, scale bar = 50 μm) and quantification of the relative fluorescence intensity (right, *n* = 6). **(D)** qPCR analysis of the NOX1, NOX2 and NOX4 mRNA levels in the aorta (*n* = 6). **(E)** Analysis of the MDA concentration and GSH levels in the sera of mice from each group (*n* = 6). The data are expressed as the mean ± SD, and n represents the number of samples. **p < 0.01 and ***p < 0.001 versus saline; ^#^ *p* < 0.05, ^##^ *p* < 0.01 and ^###^ *p* < 0.001 versus Ang II + IgG; ^§^ *p* < 0.05, ^§§^ *p* < 0.01 and ^§§§^ *p* < 0.001 versus Ang II + anti-VCAM-1 (0.1 mg).

Aortic infiltration of macrophages is a hallmark of hypertension. We next evaluated the effect of the VCAM-1 antibody on the infiltration of macrophages in the aorta. Immunohistochemical staining with a Mac-2 antibody revealed that compared with the mice cotreated with saline and IgG control, the mice that received Ang II infusion showed an increased Mac-2-positive area in the aorta and that this increase was dose-dependently reduced in the VCAM-1 antibody-treated mice (Figure 3A). Similarly, the Ang II infusion-induced upregulation of the expression of proinflammatory cytokines, including IL-1β, TNF-α and MCP-1, in the aorta was also markedly inhibited by VCAM-1 antibody administration in a dose-dependent manner (Figure 3B).

Monocytes/macrophages are the main sources of ROS, which often lead to endothelial injury (Wang et al., 2016; Lang et al., 2020). We next evaluated the impact of the VCAM-1 antibody on vascular oxidative stress. DHE staining revealed that ROS production was increased in Ang II-infused mice compared with saline control-treated mice, whereas a lower ROS level was observed in the VCAM-1 antibody-treated mice (Figure 3C). Furthermore, compared with IgG control, the VCAM-1 antibody dose-dependently reduced the Ang II-induced upregulation of the expression of NOX subunits (NOX1, NOX2 and NOX4) in the mouse aorta (Figure 3D). Moreover, the serum MDA level was lower but the serum GSH level was higher in the aortas of VCAM-1 antibody-treated mice than in those of IgG-treated mice (Figure 3E). However, high dose of anti-VCAM-1 antibody (0.2 mg) displayed a remarkable prevention of Ang II-induced inflammation and oxidative stress compared with low dose of anti-VCAM-1 (0.1 mg) (Figures 3A–E).

### Neutralization of VCAM-1 Ameliorates Ang II-Induced Vascular Endothelial Dysfunction

**FIGURE 4:**
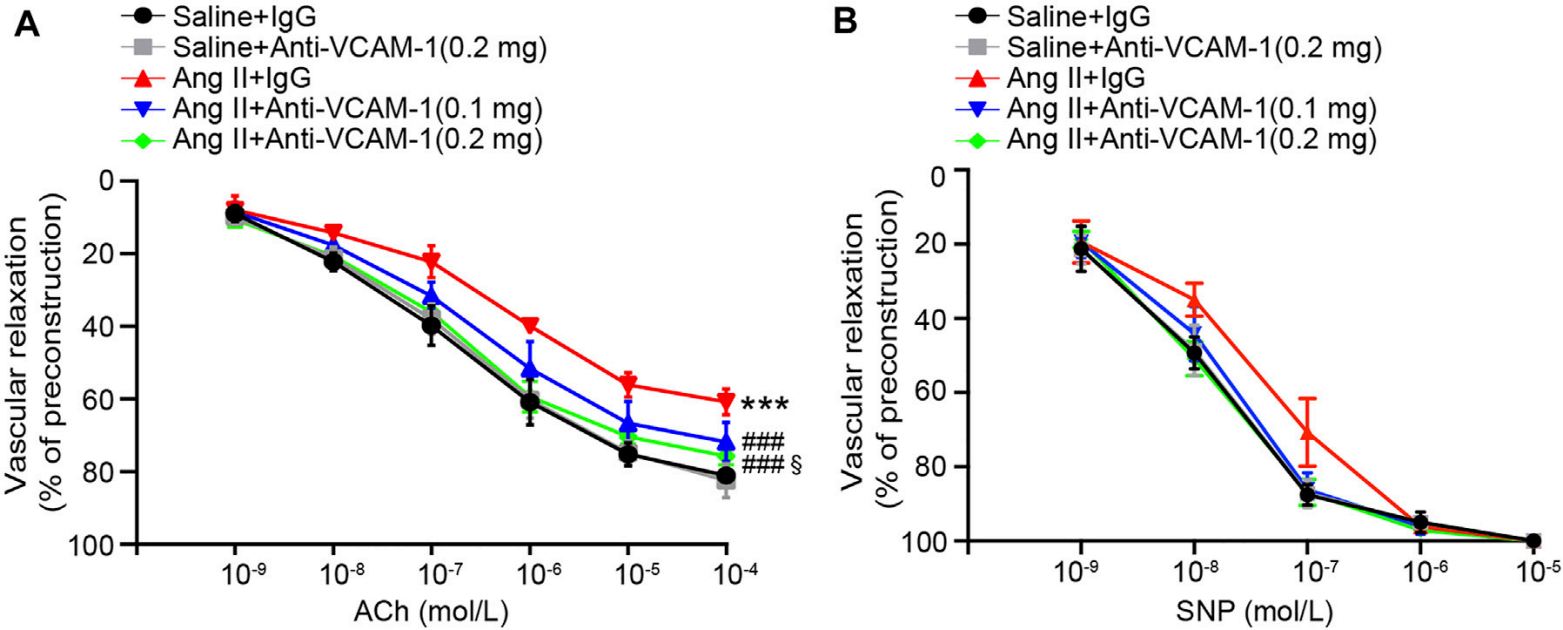
Blockade of VCAM-1 ameliorates Ang II-induced vascular dysfunction. **(A)** WT mice were treated with a VCAM-1 neutralizing antibody (0.1 and 0.2 mg/mouse) or IgG control and infused with Ang II (490 ng kg^−1^ min^−1^) or saline for 14 days. Analysis of endothelium-dependent vasodilation of the aorta in response to ACh (*n* = 6). **(B)** Analysis of endothelium-independent vasodilation of the aorta in response to SNP (*n* = 6). The data are expressed as the mean ± SD, and n represents the number of samples. ***p < 0.001 versus saline; ^###^ *p* < 0.001 versus Ang II + IgG; ^§^ *p* < 0.05 versus Ang II + anti-VCAM-1 (0.1 mg).

Inflammatory and oxidative stress frequently result in vascular endothelial dysfunction (Wang et al., 2016; Lang et al., 2020). Therefore, we assessed whether neutralization of VCAM-1 prevents Ang II-induced vascular dysfunction by analyzing isolated aortic rings. Compared with the saline control treated, Ang II infusion substantially impaired endothelium-dependent vasodilation induced by ACh, and this effect was reversed by the VCAM-1 antibody in a dose-dependent manner (Figure 4A). However, Ang II did not significantly alter endothelium-independent vasodilation in response to SNP (Figure 4B). Notably, high dose of anti-VCAM-1 antibody (0.2 mg) significantly improved Ang II-induced impairment of endothelium-dependent vasodilation compared with low dose of anti-VCAM-1 (0.1 mg) (Figure 4A).

### Neutralization of VCAM-1 Inhibits Macrophage Adhesion and Migration *in vitro*

**FIGURE 5:**
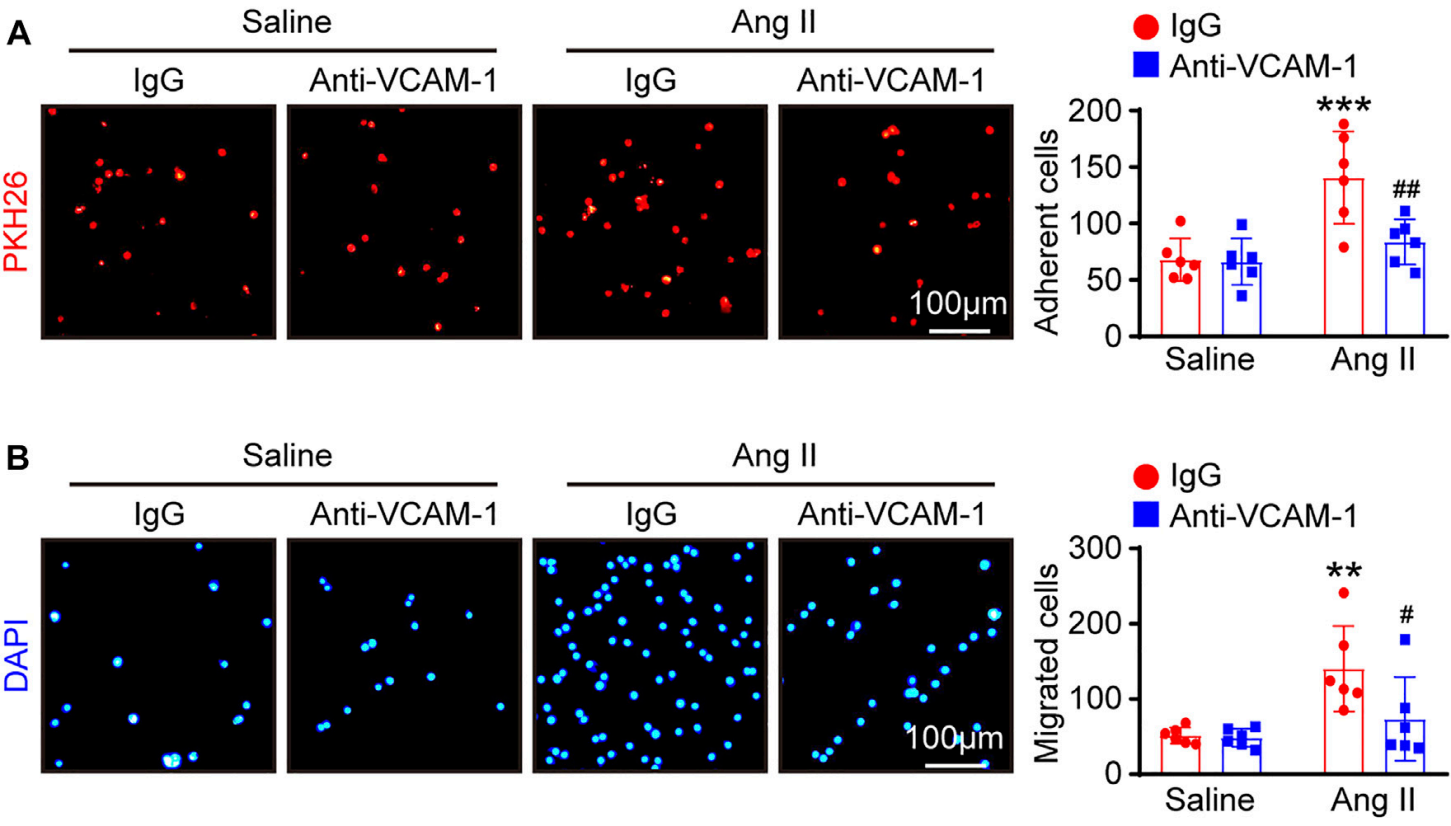
Blockade of VCAM-1 suppresses Ang II-induced macrophage adhesion and migration to HUVECs *in vitro*. **(A)** HUVECs were pretreated with anti-VCAM-1 (5 μg/ml) or IgG control for 4 h and then stimulated with saline or Ang II (100 nM) for 24 h. PKH-26 staining of macrophages (left, scale bar = 100 μm) and analysis of adherent cells (right, *n* = 6). **(B)** HUVECs were treated as described in **(A)**. Macrophages were added to the upper chamber, and HUVEC-conditioned medium was added to the lower chamber of a Transwell plate. DAPI staining of macrophages (left, scale bar = 100 μm) and analysis of migrated cells (right, *n* = 6). The data are expressed as the mean ± SD, and n represents the number of samples. **p < 0.01 and ***p < 0.001 versus saline; ^#^ *p* < 0.05 and ^##^ *p* < 0.01 versus Ang II + IgG.

VCAM-1 plays an essential role in mediating the infiltration of leukocytes into tissues (Takamatsu, 2018). We therefore examined whether the VCAM-1 antibody could alter macrophage adhesion and migration to HUVECs *in vitro*. Compared with saline control treatment, Ang II stimulation significantly enhanced the number of PKH26-labeled macrophages on the surface of HUVECs and migrated macrophages, as indicated by DAPI staining, and the VCAM-1 antibody substantially abrogated this effect (Figures 5A,B).

### Blockade of VCAM-1 Ameliorates DNA Damage and Oxidative Stress in HUVECs *in vitro*

**FIGURE 6:**
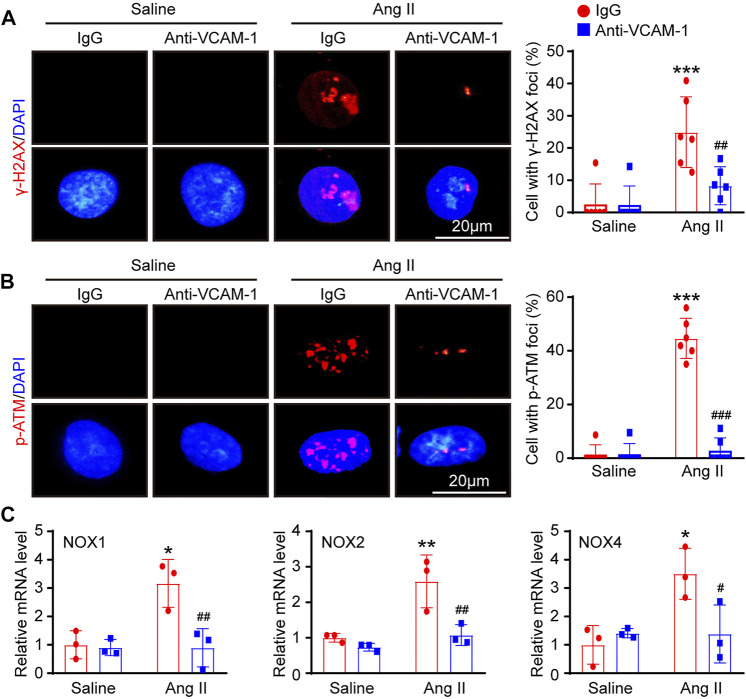
Inhibition of VCAM-1 alleviates oxidative stress in HUVECs *in vitro*. **(A)** Macrophages were pretreated with anti-VCAM-1 (5 μg/ml) or IgG control for 4 h and then stimulated with saline or Ang II (100 nM) for an additional 24 h. The conditioned supernatant was added to HUVECs. Immunofluorescence staining of HUVECs with a γ-H2AX antibody (left, scale bar = 20 μm) and quantification of γ-H2AX-positive foci (right, *n* = 6). **(B)** Immunofluorescence staining of HUVECs with a *p*-ATM antibody (left, scale bar = 20 μm) and quantification of *p*-ATM-positive foci (right, *n* = 6). **(C)** qPCR analysis of the NOX1, NOX2 and NOX4 mRNA levels in HUVECs (*n* = 3). The data are expressed as the mean ± SD, and *n* represents the number of samples. *p < 0.05, **p < 0.01 and ***p < 0.001 versus saline; ^#^ *p* < 0.05, ^##^ *p* < 0.01 and ^###^ *p* < 0.001 versus Ang II + IgG.

To further investigate whether blocking VCAM-1 inhibits DNA damage and oxidative stress in HUVECs *in vitro*, we first performed immunofluorescence staining with antibodies against histone γ-H2AX (a sensitive marker of DNA damage) and *p*-ATM. Immunostaining revealed that compared with saline control administration, Ang II treatment markedly elevated the expression of γ-H2AX and *p*-ATM in the nuclei of HUVECs and that VCAM-1 antibody treatment effectively inhibited this change (Figures 6A,B). Accordingly, compared with IgG control treatment, VCAM-1 antibody treatment attenuated the Ang II-induced upregulation of NOX1, NOX2 and NOX4 mRNA expression in HUVECs (Figure 6C).

## Discussion

**FIGURE 7:**
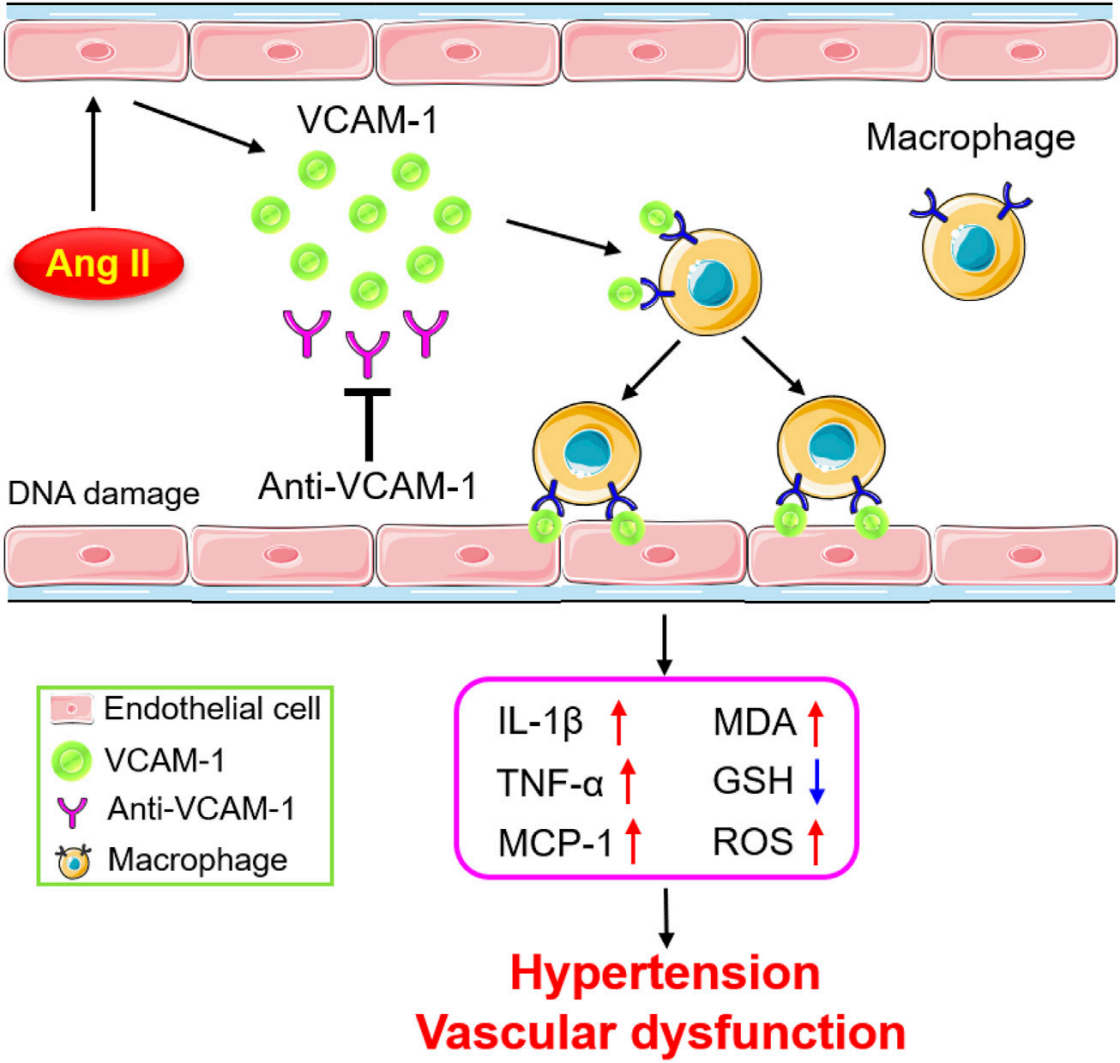
Schematic diagram of the mechanism by which VCAM-1-mediated macrophage adhesion and migration alleviate Ang II-induced hypertension and vascular remodeling. Ang II stimulates vascular ECs to release VCAM-1, which promotes the adhesion of macrophages to the arterial endothelium and their subsequent transmigration into the vascular wall. Activated macrophages secrete abundant cytokines and ROS, leading to vascular dysfunction and hypertension. In contrast, blockade of VCAM-1 significantly inhibits these effects.

The current study reveals for the first time that VCAM-1 blockade ameliorates Ang II-induced hypertension and vascular dysfunction in mice. VCAM-1 expression is significantly upregulated in the aorta after Ang II infusion, promoting the recruitment and adhesion of monocytes to the vascular endothelium. Activated macrophages produce large amounts of proinflammatory cytokines and ROS, resulting in vascular endothelial dysfunction and hypertension. In contrast, inhibiting VCAM-1 markedly prevents these effects (Figure 7). Therefore, blockade of VCAM-1 represents a novel therapeutic option for hypertension.

VCAM-1, a cell adhesion molecule (CAM), is a transmembrane type I protein that is mainly expressed on ECs during inflammation (Cook-Mills et al., 2011). Previous studies have suggested that VCAM-1 plays an essential role in regulating the inflammatory response and the development of related diseases by mediating the leukocyte adhesion to and infiltration into tissues (Takamatsu, 2018). VCAM-1 gene expression is regulated by multiple transcription factors and cytokines, including NF-kB, Ap-1, SP-1, IL-1β, and TNF-α (Cook-Mills et al., 2011; Lee et al., 2014). For example, Ang II upregulates VCAM-1 expression on the EC surface through NF-kB during early plaque formation in atherogenesis (Zhang et al., 2013). Abnormal expression of VCAM-1 has been associated with various CVDs, including hypertension, cardiac remodeling, ischemic disease, atherosclerosis, and AF (Troncoso et al., 2021). It has been reported that circulatory VCAM-1 levels are higher in older men with uncomplicated essential hypertension than in their normotensive peers and are positively correlated with SBP (DeSouza et al., 1997). However, adequate antihypertensive therapy decreases sVCAM-1 levels, suggesting that VCAM-1 could be a biomarker of endothelial dysfunction in patients with hypertension (Chukaeva et al., 2018). Furthermore, Ang II infusion significantly increases VCAM-1 expression in the aortas of mice and in patients with persistent and paroxysmal AF, whereas the VCAM-1 level decreases after administration of the histone deacetylase selective inhibitor PCI34051 or the Ang II receptor antagonist olmesartan (De Ciuceis et al., 2005; Goette et al., 2008; Kee et al., 2019), suggesting a role for VCAM-1 in promoting AF development. In our study, we found that Ang II infusion markedly upregulated VCAM-1 mRNA and protein expression in the mouse aorta and serum (Figure 1), indicating that an increase in VCAM-1 expression may be involved in the pathogenesis of hypertension and vascular dysfunction.

Numerous experimental and clinical studies have shown that inflammation plays a critical role in the pathogenesis of hypertension (Norlander et al., 2018). Immune cells, particularly monocytes/macrophages, are involved in the pathogenesis of multiple CVDs, including hypertension (Drummond et al., 2019). Experimental data have indicated that Ang II infusion increases the number of arterial macrophages in WT mice, whereas selective ablation of lysozyme M-positive (LysM) myelomonocytic cells markedly reduces Ang II-induced hypertension, the infiltration of monocytes/macrophages and the production of superoxide in the aorta (Wenzel et al., 2011). Furthermore, LysM^+^ macrophages are important for Ang II-induced arterial inflammation, the recruitment of NK cells and local IFN-γ production (Kossmann et al., 2013). Our previous studies further demonstrated that Ang II-induced upregulation of the expression of the chemokine CXCL1 promotes the recruitment of CXCR2^+^ monocytes/macrophages to the vascular endothelium. These cells then release a large amount of proinflammatory cytokines and ROS, leading to hypertension and vascular dysfunction (Wang et al., 2016). In addition to chemokines, ICAM-1 also plays an important role in hypertension development. Ang II infusion upregulates ICAM-1 expression, promoting LFA-1^+^ macrophage adhesion to the endothelium and leading to the production of multiple proinflammatory cytokines and ROS, resulting in hypertension (Lin et al., 2019; Lang et al., 2020). During the early stages of atherosclerosis, an inflammatory environment can induce VCAM-1 expression, which promotes the activation and adhesion of leukocytes, resulting in dysfunctional vasculature (Cybulsky et al., 2001). The present study further demonstrated that the Ang II-induced elevation of SBP and vascular remodeling and dysfunction in mice are dose-dependently attenuated by a VCAM-1 neutralizing antibody (Figures 2–4), demonstrating that VCAM-1 contributes to the development of hypertension and vascular dysfunction induced by Ang II infusion.

Experimental data have shown that VCAM-1 plays an essential role in inflammatory cell adhesion and migration in various CVDs. VCAM-1 was the first identified adhesion molecule in rabbit and mouse atherosclerotic lesions and is highly expressed principally by ECs and intimal cells in early-stage tumors and more advanced lesions, respectively (Iiyama et al., 1999). Furthermore, blockade of VCAM-1 *via* perfusion of the isolated carotid artery was found to significantly alleviate VLA-4-dependent monocyte rolling and adherence to the early atherosclerotic endothelium in mice (Huo et al., 2000). VCAM-1 has been reported to mediate hematopoietic stem cell recruitment to the injury site through the VLA-4 pathway in mice during partial hepatic ischemia–reperfusion (IR) injury, and this effect can be abrogated by blocking VCAM-1 (Kavanagh et al., 2010). In our hypertension model induced by Ang II and *in vitro* study, we showed that the increase in the number of macrophages and the adhesion and migration of these cells to the endothelium are significantly inhibited by a VCAM-1 neutralizing antibody in a dose-dependent manner (Figure 3, Figure 5). Endothelial oxidative stress often results in the overproduction and release of ROS, which represent one of the major causes of CVDs as well as diseases associated with other tissues and organs in mice (Zhu et al., 2012). Our previous data demonstrated that Ang II stimulation significantly upregulates the expression of multiple proinflammatory cytokines (IL-1β, IL-6 and TNF-ɑ) in bone marrow-derived macrophages (Wang et al., 2016; Lang et al., 2020), which then stimulate the expression of key transcription factors, such as NF-kB and AP-1, and increase NOX1, NOX2 and NOX4 expression and the production of ROS in ECs, resulting in EC damage and dysfunction (Charoensaensuk et al., 2021; Lian et al., 2021). Consistent with these findings, the current study also showed that compared with saline control treatment, Ang II infusion markedly increases the expression levels of the proinflammatory cytokines IL-1β, TNF-α and IL-6 (Figure 3B), which supports the conclusion that Ang II stimulated macrophages to release these factors, which were responsible for the endothelial ROS response, in this experiment. Moreover, the administration of resveratrol effectively protects intestinal integrity; alleviates intestinal inflammation and oxidative stress by inhibiting the activity of SOD, GSH-Px, and CAT; and decreases MDA levels in the jejunal mucosa in diquat-challenged piglets (Xun et al., 2021). Moreover, VCAM-1 can promote both intracellular and extracellular calcium flux and specifically activate the Rho family GTPase Rac1, which results in NOX activity and ROS production (Cook-Mills et al., 2004). Our current *in vivo* and *in vitro* data indicate that blockade of VCAM-1 can dose-dependently attenuate Ang II-induced overproduction of ROS and endothelial oxidative stress (Figure 3, and Figure 6). These results support the view that inhibition of VCAM-1 plays a protective role in hypertension and vascular remodeling.

In addition, the results of the current study indicate that injection of a low dose of an anti-VCAM-1 neutralizing antibody alleviated Ang II-induced hypertension, attenuated arterial remodeling, and improved vascular dysfunction, but only to a modest degree when compared with injection of a high dose of an anti-VCAM-1 neutralizing antibody (0.2 mg). Thus, the high dose of anti-VCAM-1 neutralizing antibody (0.2 mg) may provide the better choice for the effective therapeutic dose of clinical patients.

There are also some limitations to this study. The beneficial effects of the anti-VCAM-1 antibody need to be confirmed in VCAM-1 knockout mice. Additionally, there is no evidence demonstrating whether the anti-VCAM-1 antibody are has similar effects in female mice. Finally, VCAM-1-induced vascular dysfunction in resistant vessels such as the mesentery needs to be characterized in future studies.

In conclusion, our data demonstrate for the first time that VCAM-1 activity is involved in Ang II-induced arterial hypertension and vascular dysfunction. VCAM-1 blockade can prevent the Ang II-induced hypertensive response, likely through inhibiting macrophage adhesion and migration and reducing proinflammatory cytokine and ROS levels. Therefore, selective inhibition of VCAM-1 may be a promising therapeutic approach for hypertension.

## Data Availability

The datasets presented in this study can be found in online repositories. The names of the repository/repositories and accession number(s) can be found in the article/Supplementary Material.
